# Progress in Surgery

**Published:** 1899-05-20

**Authors:** 


### PROGRESS IN SURGERY.

### SURGERY OF THE SPINE.

**Spina Bifida.**—The open method of treatment for spina bifida by incision and ligature of the neck of the sac is gradually displacing the older methods by acupuncture and injection of irritants. The advantage, also, of closing the gap in the vertebral column is everywhere being recognised. Pearson[1] reports a case in which he successfully carried out both of these procedures in a child suffering from a large lumbar spina bifida. Regarding the operation, he concludes: (1) That the patient should lie on the side with the head low; (2) the first incision should be lateral, and along the uppermost edge of the swelling to avoid injury to nerves; (3) the fluid should, so far as possible, be retained in the sac, or replaced by irrigation while the nerve cords are being separated; (4) a sponge may be introduced to prevent leakage; (5) liberating lateral incisions are made to facilitate the aponeuroses gliding over the gap in the bone; (6) a small tube should be left between the dura mater and the aponeurosis for a few days; and (7) paralysis is not necessarily a contra-indication to operation. McGregor[2] has reported two cases, one in the upper cervical region, and the other in the lumbar region, in which a similar operation was successful; and Nicol[3] one in which it was only possible to remove portions of the sac.

One unusual case of spina bifida occulta is reported by MacLulich[4] in which the following points of interest were present: (1) Non-union of the laminæ of practically the whole vertebral column; (2) yet the only evidence of spina bifida was in the region from the fourth dorsal to the second lumbar spines, and this was nothing more than a depression of the skin; (3) there was a small fistulous opening in this depressed area, which communicated with the central canal of the spinal cord; and (4) there were no other deformities, and the tuft of hair which usually grows over spina bifida occulta was absent.

Beck has found the X-rays helpful in the diagnosis of one case of spina bifida.

**Injuries of the Spine.**—Prewitt[5] contributes some interesting remarks apropos of a case of gunshot injury of the spine. From a study of his own and cases collected from literature he comes to the following conclusions: (1) That it is the duty of the surgeon to advise immediate operation in all cases of gunshot wounds of the spine, provided the wound has involved the posterior and lateral parts of the spine at an accessible part, unless the condition of the patient is such as to indicate clearly that he is hopelessly stricken. (2) To wait to see whether nature is competent to restore the damage is to wait until irreparable damage has been done in many cases—rapid degenerative changes, meningitis, and myelitis. The delay permits of the continuance of conditions the removal of which is the purpose of the operation. These considerations apply with greater force, if possible, in gunshot injuries than others. (3) The presence of complications due to penetration of the great cavities and injury of viscera will influence the question of operation, but not necessarily forbid it. Abstracts of twenty-five collected cases are appended to this paper.

[1] Brit. Med. Jour., Nov. 3, 1898. [2] Glasgow Med. Jour., Oct. 1898. [3] Ibid. [4] Brit. Med. Jour., Dec. 24, 1898. [5] Annals of Surgery, Aug., 1898.

### GYNÆCOLOGY.

**The Operative Treatment of Uterine Fibroids.**—During the last few years the surgical treatment of fibroid disease of the uterus has undergone considerable change. Until comparatively recently both patients and the bulk of the medical profession were more or less opposed to active interference in these cases for the following reasons: (1) That fibroids are not necessarily fatal if left alone; (2) they can be made to shrink by the use of ergot and similar drugs; (3) they naturally tend to decrease in size or disappear at the menopause; and (4) surgical interference, if it is to be effective, imports extreme risk to life. The relative value of the first three reasons, as Stanmore Bishop[1] points out, depends very greatly upon the amount of force which is attached to the fourth, and as the mortality from recent statistics has been shown to be small, the relative importance of the reasons for urging operation has become much greater. Reviewing the different operative measures practised at the present time, he points out that Tait's operation of removal of ovaries and tubes, although safe, and followed in many cases by decrease in size of the tumour, is open to many objections. In some cases the tumour not only does not decrease in size, but goes on enlarging, and especially is this the case in fibro-cystic tumours. In many of the worst cases, those in which surgery was most imperatively needed, it was often found that while it might be possible to remove one ovary and tube it was well-nigh impossible while the tumour remained to reach the second; and, the essential of success being the total removal of all ovarian and even tubal tissue up to the uterine cornu, the operator might find after all that his work was useless. The opinion, meanwhile, was gaining ground that the ovaries, like the ductless glands, were essential to the general equilibrium, so that this operation was open to the double objection that, while it could not be relied upon absolutely to get rid of the tumour and its results—loss of blood and pressure effects—it removed organs which might be perfectly healthy, and which were necessary to the good and satisfactory after-condition of the patient. Reviewing myomectomy or removal of the fibroid *per se*, he points out that great difficulty is sometimes found in closing the resulting cavity safely. Uterine tissue is extremely difficult to suture satisfactorily; it is not always the same, it contracts and dilates, and a suture which is firm at the moment of insertion may be anything but firm after the abdominal wall has been closed. Fibromyomata are also so often multiple, that even if they were apparently all removed one could not be sure that such growths might not start afresh in a uterus predisposed to fibrous degeneration. Turning now to the removal of the uterus itself, with its contained growths, he says that the methods adopted have followed almost precisely the same route as that previously taken by ovariotomy, extraperitoneal treatment of the pedicle being almost universally adopted at first, which, during the last few years, has been steadily giving way to the more ideal intraperitoneal operation. He thinks that of these different operative procedures that of entire removal of the uterus cervix and all—panhysterectomy—probably gives the best results, and he quotes a number of recent statistics in support of this view. He advocates leaving the tubes and ovaries unless they are irremediably diseased. The advantages of such an operation are many; by the total removal of the fibroid uterus you get rid at once and for all of the exhausting hæmorrhage, the sense of weight and discomfort, the pressure upon the bladder and rectum, the pressure upon the sacral and lumbar nerves, the obstruction to free circulation in the lower extremities, you leave behind nothing which can either start fresh growth or act as a nidus for suppuration, and you do leave behind the all-important ovarian tissue. The cardinal objection is, of course, that you take away the possibility of pregnancy, which applies also to all methods by means of which the supravaginal portion of the uterus is removed; but this objection is not worth so much as, *à priori*, it appears to be. Johnson says that during pregnancy and labour with fibroids one-third of the mothers, and more than one-half of the children, die; pregnancy, therefore, instead of being a condition for the sake of which a patient is to be advised to suffer anything, is really one of the complications most to be dreaded. While the operation for removal was associated with a high mortality it followed as a matter of course that the alternative miseries attendant upon the presence of fibroid disease were ignored or depreciated as much as possible. With the decrease of danger from operation these may be estimated at something more like their actual value and importance. He then goes on to describe the daily miseries of these patients, and says that often it is only after years of patient and almost hopeless endurance that surgery is at last invoked, and can it be wondered at that sometimes operation is under such circumstances followed by fatal results. What different prospects would surgery have if it were allowed to act whilst the patient's strength was unsapped, and whilst yet there was a reasonable amount of blood in her body.

**Torsion of the Pedicle of Ovarian Tumours.**—Mundé,[2] writing on this subject, says that the two conditions essential to the production of a torsion of the pedicle of an ovarian tumour are (1) a long slender pedicle, (2) a small tumour not larger than a fist, or at most a cocoa-nut; further, that the tumour must lie in the abdominal cavity, for the freedom of motion necessary to torsion of the pedicle is not present when the tumour lies in the pelvic cavity. What particular agencies are to blame for the rotation of an ovarian tumour is not generally known; most likely the peristaltic movements of the intestines and their frequent gaseous distension play a prominent part; probably accidental jars and shocks or a lateral recumbent position of the woman assist in the process, or the displacement of the tumour by the gradual growth of a pregnant or fibroid uterus, and the sudden emptying of a pregnant uterus. The torsion may be either gradual or sudden, either partial or total. The pedicle may be twisted once on itself or several times, until it has become so thin as to appear on the point of breaking. As a result of this interference with the blood supply to the tumour and the resulting œdema, the tumour undergoes a rapid increase in size, and the colour of its walls changes from a pearly grey to brown or black. Usually but one twist takes place at a time, each being attended with more or less acute pain on the affected side of the sub-umbilical region, by possible faintness and collapse, by moderate distension and tenderness of the abdomen, and by some increase of pulse and temperature. The symptoms, Mundé points out, are not unlike those of an acute attack of appendicitis, especially when reflex peritoneal irritation, as shown by bilious vomiting, is present, and the pain and distension happen to be on the right side. He sums up the possible results of torsion of the pedicle as follows: (1) The greater or lesser interference with the circulation and nutrition of the tumour; (2) its increase in size from serous or bloody exudation; (3) adhesions to omentum, intestines, abdominal wall, or bladder; (4) gangrene and rupture of the sac if the tumour is cystic, provided the adhesions do not save the tumour from this fate by supplying the nutrition which has been cut off through its natural channel—the pedicle; (5) chronic peritonitis, with turbid serous exudation, or, if the gangrenous cyst actually ruptures, acute septic peritonitis and death.

[1] Lancet, Jan. 28, 1899, Stanmore Bishop. [2] New York Med. Jour., Feb. 25, 1899, Mundé.

